# A Thermoresponsive Chitosan/β-Glycerophosphate Hydrogel for Minimally Invasive Treatment of Critical Limb Ischaemia

**DOI:** 10.3390/polym13203568

**Published:** 2021-10-16

**Authors:** Caroline Herron, Conn L. Hastings, Clodagh Herron-Rice, Helena M. Kelly, Joanne O’Dwyer, Garry P. Duffy

**Affiliations:** 1 Tissue Engineering Research Group, Department of Anatomy, Royal College of Surgeons in Ireland (RCSI), D02 YN77 Dublin, Ireland; cherron@rcsi.ie (C.H.); connhastings1@gmail.com (C.L.H.); helenakelly@rcsi.ie (H.M.K.); 2 School of Psychology, University College Dublin, D04 V1W8 Dublin, Ireland; herronrc@tcd.ie; 3 School of Pharmacy and Biomolecular Sciences, Royal College of Surgeons in Ireland (RCSI), D02 YN77 Dublin, Ireland; 4 Anatomy and Regenerative Medicine Institute (REMEDI), School of Medicine, College of Medicine, Nursing and Health Sciences, National University of Ireland Galway (NUIG), H91 TK33 Galway, Ireland; 5 Trinity Centre for Bioengineering, Trinity College Dublin (TCD), D02 PN40 Dublin, Ireland; 6 SFI Centre for Research in Medical Devices (CURAM), NUIG & RCSI, H91 W2TY Galway, Ireland; 7 SFI Advanced Materials and Bioengineering Research Centre (AMBER), NUIG, RCSI & TCD, H91 TK33 Galway, Ireland

**Keywords:** critical limb ischaemia, hind limb ischaemia, chitosan, angiogenesis, desferrioxamine, thermoresponsive hydrogel

## Abstract

A reduction in blood supply to any limb causes ischaemia, pain and morbidity. Critical limb ischaemia is the most serious presentation of peripheral vascular disease. One in five patients with critical limb ischaemia will die within six months of diagnosis and one in three will require amputation in this time. Improving blood flow to the limb, via the administration of angiogenic agents, could relieve pain and avoid amputation. Herein, chitosan is combined with β-glycerophosphate to form a thermoresponsive formulation (chitosan/β-GP) that will flow through a syringe and needle at room temperature but will form a gel at body temperature. The chitosan/β-GP hydrogel, with or without the angiogenic molecule desferrioxamine (DFO), was injected into the mouse hind limb, following vessel ligation, to test the ability of the formulations to induce angiogenesis. The effects of the formulations were measured using laser Doppler imaging to determine limb perfusion and CD31 staining to quantify the number of blood vessels. Twenty-eight days following induction of ischaemia, the chitosan/β-GP and chitosan/β-GP + 100 µM DFO formulations had significantly (*p* < 0.001 and *p* < 0.05, respectively) improved blood flow in the ischaemic limb compared with an untreated control. Chitosan/β-GP increased vessel number by 1.7-fold in the thigh of the ischaemic limb compared with an untreated control, while chitosan/β-GP + 100 µM DFO increased vessel number 1.8-fold. Chitosan/β-GP represents a potential minimally invasive treatment for critical limb ischaemia.

## 1. Introduction

Smart hydrogels that respond to physiological stimuli such as temperature, pH or the presence of specific enzymes hold promise as agents for site- or time-specific delivery of therapeutic molecules, which may revolutionise drug delivery. Recently smart hydrogels have been reported to facilitate on-demand drug delivery, without a burst release effect, with applications in cancer therapy and treatment of infection [1,2,3]. Herein, the therapeutic potential of a smart hydrogel in the treatment of critical limb ischaemia is investigated. Patients with critical limb ischaemia (CLI), the most severe presentation of peripheral arterial disease, have blockages in their blood vessels reducing blood flow, typically to the lower limbs [4,5,6,7]. This reduced blood flow to the tissue in the affected limb causes cell death, affecting tissue viability [4,6,7,8]. Affected patients can develop chronic ischaemic rest pain, gangrene or ulcers [4,6,7,8]. CLI has a prevalence of 1.3% in the US and an associated treatment cost of $12 billion annually on Medicare alone [6,8]. CLI is associated with significant morbidity and mortality [6]. Six months following initial diagnosis, 20% of patients will have died, while 35% will require amputation [4,5,6]. Almost 40% of patients will die within two years of amputation, while 17% will have complications requiring reamputation [4,9]. The incidence of CLI is expected to rise as the population ages [6]. Reconstructive interventions such as surgical revascularisation or transluminal angioplasty are therapeutic options but are unsuitable for many patients with co-morbidities or issues with vascular anatomy [4,5].

Therapeutic angiogenesis, induction of angiogenesis at the ischaemic site to overcome issues with blood flow, might improve tissue perfusion [5,8]. Three approaches to angiogenesis are the primary focus of past and present clinical trials: delivery of genes encoding angiogenic proteins, delivery of angiogenic proteins themselves or delivery of stem cells [8,10,11,12,13]. Growth factors used include vascular endothelial growth factor (VEGF), fibroblast growth factor-1 (FGF-1) and hepatocyte growth factor (HGF) [5,14,15,16]. Despite some promising initial results from these clinical trials of cells and growth factors, most have failed to meet their primary end-points, preventing translation to clinical use [5,14,17]. Potential reasons for this lack of success include sub-optimal cell transfection in vivo with gene therapies, the short in vivo half-life of angiogenic proteins, and lack of clarity on the optimal cell type and source to use [14,18]. Herein, we attempt to overcome these issues via the use of a smart thermoresponsive chitosan/β-glycerophosphate (β-GP) hydrogel loaded with the angiogenic small molecule desferrioxamine (DFO).

Hydrogels have previously been described for use in CLI, either as delivery vehicles for angiogenic factors, or for their own inherent angiogenic potential. Injection of a biodegradable gelatin hydrogel has previously been used to deliver the angiogenic protein FGF-1 to the gastrocnemius muscle in a phase II clinical trial. No safety issues were identified in the seven patients involved and six of the seven patients had an improvement in their six-minute walk test distance [19]. Similarly, an elastin-like recombinamar hydrogel improved angiogenesis in a murine hind limb ischaemia model [20]. The hydrogel described herein is chitosan-based. Chitosan is a natural biomaterial. It is biocompatible, biodegradable, anti-microbial and it is licensed for use as a haemostatic agent in humans [21]. The effect of chitosan on angiogenesis is a complex one. Chitosan has been used as a component of many biomaterial delivery formulations for angiogenic agents and for wound healing applications where angiogenesis is required [22,23,24,25,26]. Incorporation of chitosan into a collagen matrix significantly increased the number of vessel-like structures in vitro and improved endothelial cell recruitment compared with collagen alone in a mouse subcutaneous in vivo model [27]. However, some studies have indicated chitosan may not be angiogenic, although this may depend on the type of chitosan used, the other constituents of the formulation and the model used for testing [28,29].

The chitosan/β-GP hydrogel described in this manuscript is thermoresponsive—i.e., it is a liquid at temperatures less than 37 °C, and forms a semisolid hydrogel at 37 °C, when injected into the body [30]. Chitosan/β-GP hydrogels have been identified to have excellent potential as delivery vehicles for biomedical applications, allowing minimally invasive administration, prior to site-specific and potentially sustained delivery of therapeutics [31]. A thermoresposive methylcellulose hydrogel has previously been used to deliver stem cells in a mouse hind limb ischaemia model, although the thermoresponsive nature in that case was to facilitate site specific release of the stem cells, not minimally invasive administration [32]. The chitosan/β-GP hydrogel herein will be loaded with DFO.

DFO was first characterised in the 1950s and is used clinically as a chelation agent for iron removal in the treatment of iron overloads including acute overdose and thalassaemia [33]. DFO induces angiogenesis via upregulation of hypoxia-inducible factor-1 alpha (HIF-1α) [34]. This subsequently causes the release of a number of angiogenic molecules, including VEGF and platelet-derived growth factor (PDGF) [34,35,36]. DFO has been used in pre-clinical studies to promote wound healing and to enhance the tissue integration of biomaterials [34,37,38]. Previously, we have demonstrated sustained release of DFO for seven days from the chitosan/β-GP hydrogel [30]. The formulation also significantly increased VEGF expression in exposed human umbilical vein endothelial cells [30]. Herein we progress this DFO-loaded chitosan/β-GP hydrogel to in vivo studies. The in vivo model used is a murine hind limb ischaemia model. It is hypothesised that injection of the pro-angiogenic agent DFO, loaded in a thermoresponsive chitosan/β-GP formulation, will improve perfusion in a mouse model of hind limb ischaemia.

## 2. Materials and Methods

### 2.1. Materials

Adult male Balb/C mice were purchased from Harlan Sprague Dawley Inc. (Bicester, Oxfordshire, UK). Ultrapure chitosan was obtained from Pronova Biomedical, Oslo, Norway (UP CL214). The chitosan had a molecular weight of 150–400 kDa and a degree of deacetylation of >90%. β-glycerophosphate and desferrioxamine were purchased from Sigma Aldrich (Dublin, Ireland). CD31 and other reagents for immunostaining were supplied by Vector Labs (Burlingame, CA, USA). OCT and isopentane were obtained from VWR (Dublin, Ireland).

### 2.2. Animal Model

A mouse hind limb ischaemia model, based on that described by Limbourg et al., is used herein [39]. Ethical approval for the study was granted by the Health Products Regulatory Authority (HPRA), the competent authority for implementation of EU legislation Directive 2010/63/EU for the protection of animals used for scientific purposes in Ireland. The appropriate Department of Health licences were obtained by the institution (RCSI) and ethical approval from the animal research ethics committee in RCSI was also obtained.

Adult, male, Balb/C, 10-week-old athymic mice, weighing 20–30 g, were used for this study. Mice were anaesthetised for vessel ligation using a combination of isoflurane (5% induction, 2% maintenance), 33% oxygen, and 66% nitrous oxide. Pre-operative analgesia was provided via an injection of buprenorphine (0.1 mg/kg) and prophylactic antibiotics were provided (Marbocyl, marbofloxacin).

Once anaesthetised, mice were prepared and draped and placed in the supine position and microscopic surgery was performed under sterile conditions. This involved making a longitudinal incision in the left groin and dissecting through the fatty tissue layer to expose the femoral neurovascular bundle. The common femoral artery above the level of the bifurcation was identified. The common femoral artery was dissected away from the vein and nerve, controlled and ligated in continuity. A small section of the vessel was removed to create ischaemia.

Initially a pilot study was performed on mice to determine: 1: if the chitosan gel could be safely injected into the hind limb of the mouse without major complications and 2: if ischaemia could be confirmed with laser Doppler imaging (LDI). All mice underwent surgical ligation of the common femoral artery of one leg. LDI was performed on both the ischaemic limb and contra-lateral non-ischaemic limb to confirm the usefulness of LDI. The chitosan/β-GP hydrogel was injected into the de-vascularised muscle bed of the mice, and they were monitored for any ill-effects over four weeks, with no issues noted. 

**Figure 1:**
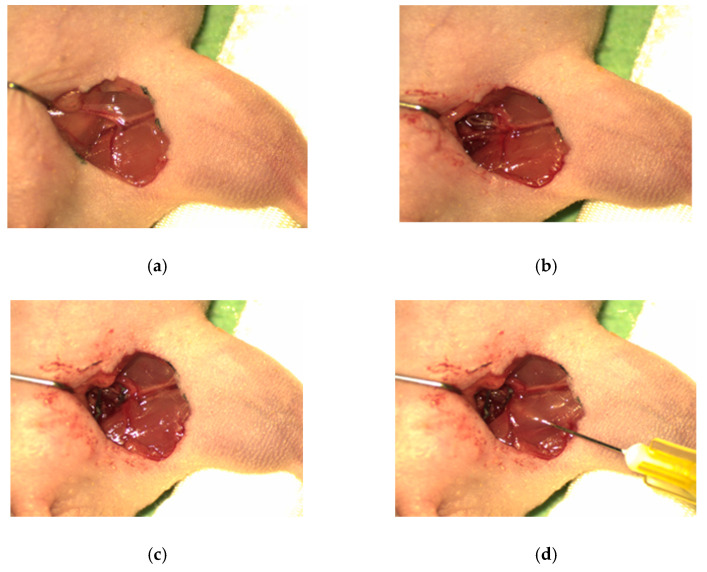
Images demonstrating the anatomy of the murine hind limb: (**a**) the vasculature at the groin, (**b**,**c**) the dissection of the femoral vessels, and (**d**) injection of the chitosan/β-glycerophosphate hydrogel into the de-vascularised muscle bed.

Following this pilot study, the main study was initiated. This study used 24 mice, 8 in each group, and the groups were as follows: control, chitosan/β-GP hydrogel alone and chitosan/β-GP hydrogel + 100 µM DFO. Mice in all groups underwent vessel ligation (Figure 1a–c). The control group received no experimental treatment following vessel ligation. Treatment groups received either 300 µL of chitosan/β-GP hydrogel alone or 300 µL of chitosan/β-GP hydrogel loaded with 100 µM DFO (Figure 1d) (Table 1). The hydrogel was injected along the de-vascularised muscle bed in 6 distinct regions immediately after ligation. The skin was closed immediately following injection with two interrupted horizontal mattress sutures using 5/0 silk suture. The mice were monitored daily, and additional doses of analgesia or antibiotics were given as required. Mice were euthanised and fixed using perfusion fixation 28 days following ligation.

### 2.3. Preparation of Thermoresponsive Chitosan/β-GP Formulations

Ultra-pure chitosan with a degree of deacetylation >95% (Pronova Biomedical, Oslo, Norway) was used in the preparation of all gels. For a typical 2% *w*/*v* chitosan, 7% *w*/*v* β-GP hydrogel, 100 mg of chitosan was dissolved in 4.5 mL dH_2_O at pH 8–9. 350 mg of β-GP was dissolved in 0.5 mL dH_2_O, also at pH 8–9, and chilled on ice. The β-GP solution was then added drop-by-drop to the chitosan solution, with stirring, on ice, to achieve a homogenous formulation. Formulations were stored on ice until use. Chitosan/β-GP loaded with 100 µM DFO was prepared in the same manner except that DFO was added to the constituent dH_2_O to give a final concentration of 100 µM.

### 2.4. Assessment of Perfusion via Laser Doppler Imaging

Laser Doppler imaging (LDI) is a non-invasive imaging technique that uses a low power He-Ne laser to scan the limbs of living animals and provide a measurement of perfusion [36]. The Doppler signal is linearly proportional to the perfusion in the upper 200–300 μm of the skin [36]. LDI (Periscan PIM 3, Perimed, Sweden) was used throughout the study, in accordance with the manufacturer’s instructions, as a marker of limb perfusion. Pre-operatively both legs were scanned and mice with a starting perfusion difference of greater than 15% between both lower limbs were excluded from the study.

During scanning, mice were placed in the prone position. The scan head was positioned between 10 and 15 cm above the mouse and a region of interest (ROI) was created over the feet (plantar sole) on both the ischaemic and control limbs. In this area skin pigmentation and hair does not influence the perfusion signal [39,40]. All scans were repeated and an average of the two scanning measurements taken. Tissue perfusion was recorded as a colour coded image and a perfusion unit value was established for each ROI. Following the ligation of the common femoral artery, the limbs were again scanned to confirm creation of ischaemia. Thereafter, at weekly time points, the mice were anaesthetised, and measurements of limb perfusion were taken. Hind limb blood flow is expressed as the percentage difference between the left (ischaemic) and the right (non-ischaemic) limbs.

### 2.5. Assessment of Ambulatory Impairment and Ischaemic Damage

A semi-quantitative assessment of the ambulatory impairment of the ischaemic limb was performed for each mouse at daily intervals. This indicated the impact of ischaemia on the mouse and the subsequent improvement or worsening of mobility following treatment. A scoring system (Table 2) was used to grade the degree of disability exhibited by the mouse in the ischaemic hind limb. In addition to a functional assessment of the limb, the degree of ischaemic damage to the limb was also graded based on the appearance of the limb (Table 3).

### 2.6. Tissue Processing

Following successful perfusion fixation of the mice 28 days after ligation and treatment, both hind limbs were removed, and the thigh and calf muscles of both hind limbs were dissected out. The four muscle groups (thigh and calf from both hind legs) were then immersed in a sucrose solution for cryoprotection. Muscles were first placed in 10 mL of 15% *w*/*v* sucrose at 4 °C for 3 h and then transferred to 10 mL of 30% *w*/*v* sucrose solution at 4 °C overnight. The muscles were then covered with optimal cutting temperature compound (OCT) and snap frozen in isopentane by suspending them in liquid nitrogen. The muscle tissue was then stored at −80 °C until processing.

### 2.7. Tissue Sectioning

A Cryostat (Leica CM 1950) was used to section 10 μm samples at −20 °C, which were placed on Thermo Scientific Menzel-Glaser Superfrost Plus slides. Each slide contained 6 tissue samples, and a total of 15 slides were prepared for each muscle from 3 different regions of the muscle. These slides were then stored at −20 °C prior to staining.

### 2.8. Tissue Staining—CD31

Three slides from each muscle group of each animal were stained with an immuoflorescent anti-CD31 antibody, using a standard protocol, as a marker of endothelial cells and, consequently, angiogenesis. Staining involved antigen retrieval and inhibition of endogenous peroxidase activity prior to incubation with the primary antibody (rabbit polyclonal to CD31) and secondary antibody (goat anti-rabbit IgG). 

### 2.9. Quantification of Capillary Density

After staining, sections were viewed at 10× magnification on a Nikon Microscope Eclipse 90i. Fifteen randomly chosen microscopic fields from three different sections in each tissue block were examined for the presence of capillary endothelial cells for each mouse specimen. Capillary density is expressed as the number of CD31-positive features per high power field.

### 2.10. Statistical Analysis

The number of animals required in each group was determined by performing a power calculation based on the following factors: 80% power, difference to be detected 5%, significance level *p* < 0.05. This yielded a required sample size of six per group. An extra two animals were added to each group to allow for potential attrition. Data were analysed using a 2-way mixed ANOVA, a 1-way non repeated measures ANOVA, and the Tukey post-hoc comparative procedure with GraphPad version 8.

## 3. Results

### 3.1. Laser Doppler Imaging: Assessment of Perfusion

**Figure 2:**
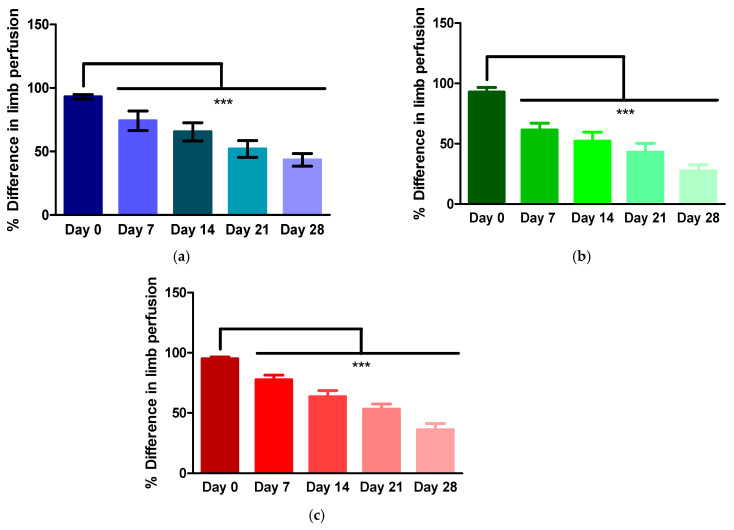
Percentage difference between ligated (ischaemic) and non-ligated limbs in the presence of (**a**) no treatment (control), (**b**) chitosan/β-glycerophosphate (GP) hydrogel alone used as a treatment on the ligated limb and (**c**) chitosan/β-GP hydrogel loaded with 100 µM desferrioxamine used as a treatment on the ligated limb. In all groups, each time point was statistically significantly different from the day 0 measurement. *** = *p* <0.001, *n* = 8.

The animals were scanned immediately post-operatively (day 0) to confirm ischaemia and again at day 7, 14, 21 and 28. At each time point, the perfusion difference between the ligated and non-ligated limb was calculated. A smaller percentage difference between the two limbs indicates blood flow improving in the ligated limb. A 0% difference would indicate perfusion in both limbs was the same. Figure 2 shows that in all groups, control (no treatment), chitosan/β-GP hydrogel alone and chitosan/β-GP hydrogel + 100 µM DFO, the perfusion difference between the two limbs reduced significantly at each time point, compared with the day 0 measurement. The percent difference in blood flow between the limbs reduced by 2.16-fold from day 0 to day 28 in the control group. In the presence of the chitosan/β-GP hydrogel, the difference in blood flow between the two limbs reduced by 3.4-fold over the 28 days of the study. Loading the chitosan/β-GP hydrogel with 100 µM DFO, resulted in a 2.64-fold reduction in perfusion difference between the limbs over the 28 days.

### 3.2. Laser Doppler Imaging: Comparison between Groups

**Figure 3:**
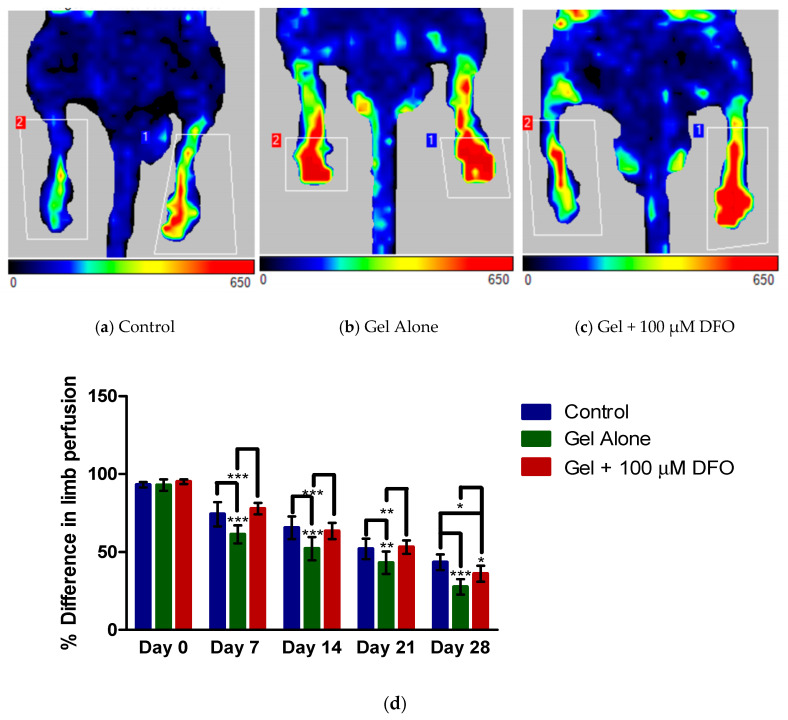
Representative images from laser Doppler imaging 28 days following ligation of limb “2” and (**a**) no treatment (control), (**b**) treatment with chitosan/β-glycerophosphate(GP) alone or (**c**) treatment with chitosan/β-GP + 100 µM desferrioxamine (DFO). (**d**) Percent difference in limb perfusion, a reduction indicating improved perfusion, at all time points, for the control (untreated), gel alone (chitosan/β-GP alone) and gel + 100 µM DFO (chitosan/β-GP + 100 µM DFO) groups. * = *p* < 0.05, ** = *p* < 0.01, *** = *p* < 0.001, *n* = 8.

Figure 3a–c shows the LDI of each group, control, chitosan/β-GP hydrogel alone and chitosan/β-GP + 100 µM DFO, 28 days following vessel ligation and/or treatment. In all cases limb “2” is the ischaemic limb, and colours towards the red end of the spectrum indicate blood flow. More blood flow occurred in limb “2” in both the chitosan/β-GP hydrogel alone and chitosan/β-GP + 100 µM DFO groups compared with the control group. Figure 3d compares the perfusion difference between the ligated and non-ligated limbs across the treatment groups at 7, 14, 21 and 28 days. At 7 days post-treatment, the chitosan/β-GP hydrogel alone had significantly improved limb perfusion compared with both the control group (no treatment) and the chitosan/β-GP hydrogel with 100 µM DFO. This trend was repeated at day 14 and at day 21. At day 28, both the chitosan/β-GP hydrogel alone and chitosan/β-GP + 100 µM DFO, significantly improved perfusion compared with the control group. At day 28, the perfusion difference between the ligated and non-ligated limbs was just 27% in the chitosan/β-GP hydrogel alone group and 36% in the chitosan/β-GP + 100 µM DFO group, compared with a perfusion difference of 43% in the control group. This indicates blood flow had recovered more in the presence of the chitosan/β-GP hydrogel and the chitosan/β-GP + 100 µM than in the untreated control group.

### 3.3. Assessment of Ambulatory Impairment and Ischaemic Damage

No mouse experienced immobility or became non-weight bearing on the ischaemic limb. One mouse, out of eight, in each group, was observed to be dragging the ischaemic limb throughout the study period. The other seven mice in each group did not exhibit dragging of the ischaemic limb. 

Visual assessment of ischaemia indicated moderate to severe discolouration in the ischaemic limb of one mouse in the chitosan/β-GP + 100 µM DFO group; no other mice showed moderate to severe discolouration of the ischaemic limb. 

### 3.4. Histology—Thigh

**Figure 4:**
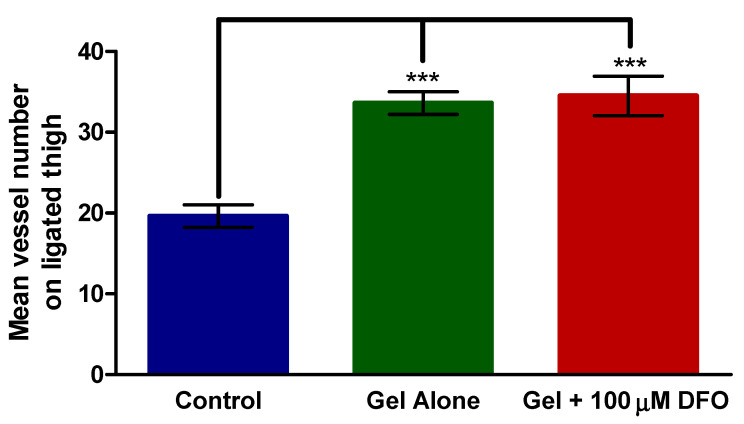
CD31+ vessel count in the thigh of the mice following euthanasia. Mice received no treatment (control), treatment with chitosan/β-glycerophosphate (GP) alone (gel alone) or treatment with chitosan/β-GP + 100 µM desferrioxamine (DFO) (gel + 100 µM DFO). *** = *p* < 0.001, *n* = 8.

Muscles from each mouse were stained for CD31 indicating the presence of endothelial cells and, by implication, blood vessels. The mean CD31+ vessel counts are displayed below in Figure 4 for the thigh of the ligated limb. In all cases, ligation significantly increased the vessel count in the limb compared with the control, non-ligated limb (Figure S1, Supplementary Materials). The chitosan/β-GP hydrogel alone and the chitosan/β-GP hydrogel + 100 µM DFO both significantly (*p* < 0.001) increased the mean vessel count compared with the control group. The chitosan/β-GP hydrogel alone increased the vessel count 1.7-fold compared with the control, while chitosan/β-GP + 100 µM DFO increased the vessel count 1.8-fold compared with the control group. There was no significant difference between the chitosan/β-GP alone group and the chitosan/β-GP + 100 µM DFO group.

### 3.5. Histology—Calf

**Figure 5:**
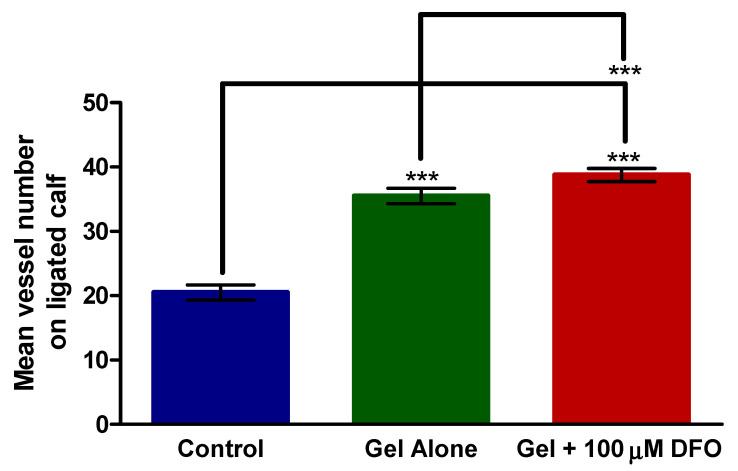
CD31+ vessel count in the calf of the mice following euthanasia. Mice received no treatment (control), treatment with chitosan/β-glycerophosphate (GP) alone (gel alone) or treatment with chitosan/β-GP + 100 µM desferrioxamine (DFO) (gel + 100 µM DFO). *** = *p* < 0.001, *n* = 8.

Figure 5 shows the CD31+ vessel count from the calf of the mice. Both the chitosan/β-GP hydrogel alone and the chitosan/β-GP hydrogel + 100 µM DFO significantly increased the vessel count compared with the control group. The chitosan/β-GP hydrogel increased the vessel count by 1.7-fold compared with no treatment. Chitosan/β-GP + 100 µM DFO increased the vessel count by 1.9-fold compared with no treatment. Chitosan/β-GP + 100 µM DFO also significantly increased vessel number in the thigh compared to the chitosan/β-GP hydrogel. In all cases the vessel number was significantly increased in the ligated limb, compared to the non-ligated limb (Figure S2, Supplementary Materials). 

## 4. Discussion

The in vivo study described aimed to replicate critical limb ischaemia through ligation of the common femoral artery in a mouse. This produced one ischaemic limb, which could be compared with the opposite limb, which had not been ligated and had normal blood flow. Laser Doppler imaging was used to confirm differences in real time perfusion in the limbs. Regardless of whether mice were treated with the chitosan/β-GP hydrogel alone, chitosan/β-GP + 100 µM DFO or given no treatment, the perfusion difference between the two limbs significantly reduced over the 28 days of the experiment, indicating some recovery of blood supply in the ischaemic limb. This indicates that there is a natural response to ischaemia which prompts collaterogenesis to supply blood to the ischaemic limb. However, this response is far from perfect at restoring blood flow. Without treatment (control group), 56% of blood flow had been restored to the ligated limb by day 28, this compares to 72% and 64% in the chitosan/β-GP alone and chitosan/β-GP + 100 µM DFO groups respectively. Thus, treatment can improve blood flow to the limbs. At day 28, both chitosan/β-GP alone and chitosan/β-GP + 100 µM DFO significantly (*p* < 0.001 and *p* < 0.05 respectively) improved blood flow compared with no treatment. This improved angiogenesis in the presence of an injectable, thermoresponsive chitosan/β-GP hydrogel has not previously been reported in the literature, although chitosan-based formulations have previously shown pro-angiogenic effects [41,42]. The addition of pro-angiogenic DFO to the chitosan hydrogel was hypothesised to increase angiogenesis more than chitosan alone. This additional improvement was not observed here. Only one dose of DFO was used here, based on the dose tested in previous in vitro studies. Further studies with higher doses of DFO may show increased angiogenesis on DFO incorporation. We previously showed this hydrogel could release bioactive DFO for seven days, further modification of the gel to produce a longer release period may improve angiogenesis [30]. However, the improvement in limb perfusion observed compares well with studies reported in the literature. Beegle and colleagues delivered mesenchymal stem cells (MSCs), engineered to over-express VEGF, to mice, in a hind limb ischaemia model [10]. Recovery of blood flow in that study, occurred mainly from six weeks onwards, and so, was much slower when compared with recovery seen in this study. At the ten-week endpoint of the study, mice treated with the modified MSCs had recovered 50% blood flow in the ischaemic limb, this contrasts with the 72% increase in blood flow at four weeks with the chitosan/β-GP hydrogel alone reported in our study [10]. Ferraro and colleagues delivered plasmid DNA encoding FGF-1 via intradermal injection followed by cutaneous electroporation, to increase cellular uptake of the plasmid [43]. At 14 days following delivery to the ischaemic hind limb of a rat, the FGF plasmid had restored blood flow in the ischaemic limb to almost 100% [43]. The angiogenic elastin-like recombinamer hydrogel reported by Marsico and colleagues achieved a perfusion ratio (ischaemic/non-ischaemic x 100) of 53 at day 21, slightly lower than the perfusion ratio of 57 we have reported for the chitosan/β-GP hydrogel alone [20]. Alginate microbeads containing HGF have also been delivered to the mouse hind limb and achieved a perfusion ratio of 41.5 nine days after treatment [44]. While a day 9 timepoint was not taken in this study, the results are in line with the ratio of 38.6 at day 7 and 47.9 at day 14 from the chitosan/β-GP alone group.

Assessing perfusion provides information on blood flow in the limb, which is important in terms of the overall effect of the formulation and potential benefit to a patient. However, the effect of the formulations on the vasculature should also be investigated. Vessel ligation increased the CD31+ vessel count in the calf and thigh of all mice. This corresponds with the perfusion data above, such that there was a physiological response to ischaemia which caused angiogenesis to occur to overcome the ischaemia. Figure 4 showed that both the chitosan/β-GP hydrogel alone and the chitosan/β-GP hydrogel + 100 µM DFO significantly (*p* < 0.001) increased the vessel count compared with the control (no treatment) group. The calf muscle groups displayed similar findings (Figure 5). Both the chitosan/β-GP hydrogel alone and the chitosan/β-GP hydrogel + 100 µM DFO significantly increased the vessel count compared to the untreated control. This corresponds with the 28-day perfusion studies with both treatments significantly improving perfusion compared to the untreated control group. The number of blood vessels in the limb injected with the FGF plasmid mentioned earlier, tested by Ferraro et al., was almost 2-fold higher than in the control group, similar to the 1.7-fold and 1.9-fold increases in vessel number seen with the chitosan/β-GP hydrogel alone and chitosan/β-GP + 100 µM DFO, respectively [43]. 

The data presented in this manuscript indicate that chitosan/β-GP may be of use to improve perfusion in the treatment of critical limb ischaemia; however, further animal studies would be required to confirm this. There are limitations to our study. Only one dose of DFO was used. This was based on in vitro studies, but significant dose optimisation may be needed for an animal model. There are also some limitations to the mouse hind limb ischaemia model. The mice are young and healthy and therefore are not directly comparable to the usually elderly, co-morbid (diabetic, hypertensive, smoker) population who develop vascular disease in the human population. Furthermore, the model delivers an acutely ischaemic limb due to the abrupt cut-off of the blood supply, whereas the limb ischaemia of peripheral arterial disease in humans is a gradual disease which brings about a reduction in blood flow over time. This progressive ischaemia allows time for the development of a collateral circulation, although it is widely accepted that this collateral circulation cannot salvage end-stage critical limb ischaemia [45,46].

Importantly, the smart thermoresponsive hydrogel presented, can be injected using a standard needle and syringe commonly used in the clinic. Thus, this formulation could represent a minimally invasive therapy for patients which can be delivered on a regular consultant visit without requiring the significant resources associated with inpatient admission and surgical procedures.

## 5. Conclusions

Laser Doppler imaging and assessment of CD31+ vessel count have been used to illustrate an increase in angiogenesis and perfusion following treatment with an injectable, thermoresponsive chitosan/β-glycerophosphate hydrogel with or without DFO. This smart hydrogel formulation has the potential to be administered easily in the clinic, to improve treatment outcomes, morbidity, and mortality in patients with critical limb ischaemia.

## Figures and Tables

**Table 1:** Treatment groups used in the hind limb ischaemia mouse study.

Group Name	Treatment
Control	None
Chitosan/β-GP gel alone	300 µL chitosan/β-glycerophosphate hydrogel
Chitosan/β-GP gel + DFO	300 µL chitosan/β-glycerophosphate hydrogel loaded with 100 µM DFO

**Table 2:** Visual scoring system used to grade the degree of disability exhibited by mice. Mice were inspected visually daily and the score is assigned based on the ischaemic hind limb.

Score	Assessment of Ambulatory Impairment of the Ischaemic Limb
0	Flexing the toes to resist traction on the tail, as for the non-operated foot
1	Plantar flexion
2	No dragging but no plantar flexion
3	Dragging of the foot
4	Non-weight bearing
5	Immobile

**Table 3:** Visual scoring system used to grade the degree of damage to the limb caused by ischaemia.

Score	Assessment of Ischaemic Damage to Limb
0	Plantar flexion, no discolouration
1	Plantar flexion, mild discolouration
2	No plantar flexion, mild discolouration
3	No plantar flexion, moderate to severe discolouration
4	Any necrosis of the foot
5	Amputation

## Data Availability

The datasets generated during and/or analysed during the current study are available from the corresponding author on reasonable request.

## Supplementary Materials

The following are available online at https://www.mdpi.com/article/10.3390/polym13203568/s1, Figure S1: CD31 vessel counts in the thigh of ligated and non-ligated limbs. Figure S2: CD31 vessel counts in the calf of ligated and non-ligated limbs.
