# Supplementary material for: A Thermoresponsive Chitosan/β-Glycerophosphate Hydrogel for Minimally Invasive Treatment of Critical Limb Ischaemia
Source: Polymers (Basel). 2021 Oct 16;13(20):3568. doi: 10.3390/polym13203568 (PMC8539345; doi:10.3390/polym13203568)
Supplement: Supplementary file 1 [file polymers-13-03568-s001.zip › polymers-1410378-supplementary.pdf]

A thermoresponsive chitosan/β-glycerophosphate hydrogel for minimally invasive treatment of critical limb ischaemia

Caroline Herron, Conn L. Hastings, Clodagh Herron-Rice, Helena M. Kelly, Joanne O'Dwyer, Garry P. Duffy.

Supplementary Material

CD31 staining of the thigh and calf of mice was performed. This staining showed that ligation of the vessel, significantly ($p<0.001$) increased the vessel count, compared to that in the non-ligated limb in both the thigh (Fig. S1) and the calf (Fig. S2).

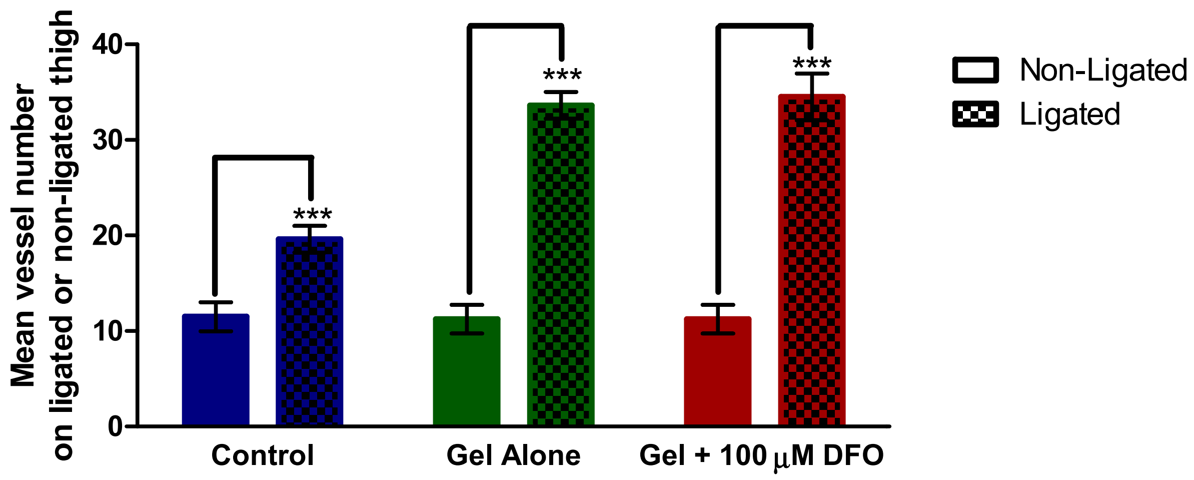

Figure S1 CD31 vessel counts in the thigh of ligated and non-ligated limbs. Ligation significantly increased the vessel count in the thigh compared to the thigh of a non-ligated limb. ***=p<0.001. n=8.

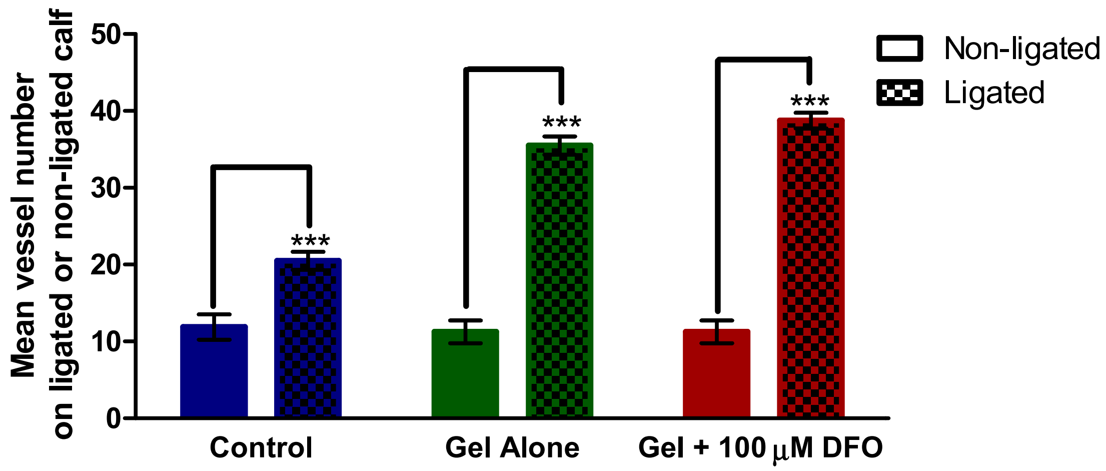

Figure S2 CD31 vessel counts in the calf of ligated and non-ligated limbs. Ligation significantly increased the vessel count in the thigh compared to the thigh of a non-ligated limb. ***=p<0.001. n=8.
